# Developing an efficient MGCR microneedle nanovaccine patch for eliciting Th 1 cellular response against the SARS-CoV-2 infection

**DOI:** 10.7150/thno.83390

**Published:** 2023-09-04

**Authors:** Ang Gao, Yunsheng Chen, Hui Liang, Xinyuan Cui, Amin Zhang, Daxiang Cui

**Affiliations:** 1 Institute of Nano Biomedicine and Engineering, Shanghai Engineering Research Center for Intelligent Instrument for Diagnosis and Therapy, School of Sensing Science and Engineering, Shanghai Jiao Tong University, Shanghai 200240, China.; 2 National Engineering Research Center for Nanotechnology, 28 East Jiangchuan Road, Shanghai 200241, China.; 3 Radiology Department of Ruijin Hospital, Shanghai Jiao Tong University School of Medicine, 197 Ruijin Second Road, Shanghai 200025, China.

**Keywords:** Receptor-Binding Domain, nanovaccine, microneedle patch, RBD-specific Type 1 cellular response, graphene oxide

## Abstract

**Rational**e**:** Novel vaccine R&D is essential to interrupt the COVID-19 pandemic and other epidemics in the future. Subunit vaccines have received tremendous attention for their low cost and safety. To improve the immunogenicity of subunit vaccines, we developed a novel vaccine adjuvant system.

**Methods:** Here we rationally designed a CpG 1018 and graphene oxide-based bi-adjuvant system to deliver the Receptor-Binding Domain (RBD) of the SARS-CoV-2 spike protein and obtained the graphene oxide-based complex adjuvant nanovaccine (GCR). Furthermore, we developed a microneedle patch vaccine (MGCR) based on the GCR vaccine.

**Results:** GCR nanovaccine displayed superb antigen loading and encapsulation efficiency. Two dosages of vaccination of GCR nanovaccine could elicit adequate RBD-specific binding antibody response with 2.14-fold higher IgG titer than Alum adjuvant vaccine. The peptide pools assay demonstrated the robust RBD-specific Type 1 Cellular response induced by the GCR nanovaccine in CD8^+^ T cells. Furthermore, we prepared an MGCR microneedle patch, which generated a similar RBD-specific binding antibody response to the GCR vaccine, sustained a high antibody level above 16 weeks, and significantly elevated the T_cm_ proportion in mouse spleen. The MGCR microneedle patch vaccine also could be stably stored at room temperature for several months and administrated without medical staff, which maximizes the vaccine distribution efficiency.

**Conclusion:** The vaccine system could significantly improve the vaccine distribution rate in low-income areas and offer a potential vaccination approach to fight against the SARS-Cov-2 infection and other pandemics occurred in the future.

## Introduction

The COVID-19 pandemic has been lasting nearly for 3 years and caused nearly 769 million people infected including 6.7 million people died around the world, according to the latest WHO reports 1. An efficient vaccine is essential to intercept the spread risk caused by emerging SARS-CoV-2 mutant strains. Up to now, there are at least 200 vaccines in clinical and preclinical development, and more than 30 vaccines were approved for urgent use 2, 3. The licensed vaccines could divide into four kinds: mRNA vaccines, Adenovirus vaccines, inactivated vaccines, and recombinant protein vaccines 4, 5.

mRNA vaccines were constructed of phospholipid vesicle and SARS-CoV-2 spike protein mRNA sequence 6-8. Due to the poor stability of mRNA, the ultracold atmosphere was essential for vaccine storage and distribution, which extremely increased the cost and limited the vaccination rates in underdeveloped areas 9, 10. Adenovirus vaccines, inactivated virus vaccines, and subunit protein vaccines have lower demand for storage environments and well-established techniques, which would be the preferred choices for developing countries 11, 12. Adenovirus (Ad) 5 and Ad 26 were the most universal virus vectors 13-15. The Convidecia (CanSino Biological) was an Ad 5 vector-based vaccine, the single dose of administration of Convidecia could elicit adequate neutralizing antibodies and T-cell responses 16. But the Ad 5 infection rate was grown with age, which induced less immunogenic in elderly people 17. Inactivated vaccines were produced by deactivated viruses through physical and chemical methods, which have huge advantages in vaccine safety, storage, and production 18-20. However, inactivated vaccines could elicit a slight T-cell response, which required larger vaccination dosages. Meanwhile, the requirement of biosafety level 3 also limited the vaccine manufacture efficiency.

Licensed subunit recombinant protein vaccines used the SARS-CoV-2 S protein RBD fragments to elicit an immune response 21-24. The RBD recombinant protein manufacture has a lower requirement of biosafety level and cost. Furthermore, the precision recombinant protein structure minimized the safety risk caused by uncertain factors 25, 26. However, recombinant protein usually has poor immunogenicity, the improvement of immunogenicity is necessary for RBD vaccine design. A direct approach is to optimize protein sequences, fragment lengths, and immunization schedules 27-29. For the urgent demand for epidemic vaccines, an optimal approach is to develop a universal adjuvant system to enhance the immunogenicity of subunit proteins 30-33. Alum adjuvant, as the wildest application adjuvant system, can prominently elicit humoral immune responses and promote the production of neutralization antibodies. But alum adjuvant could not elicit T cell-mediated immunity response 34, 35. Therefore, we recommend a novel antigen delivery system based on graphene oxide (GO).

GO has an excellent drug loading efficacy, due to its 10-folder surface area to other nanoparticles 36, 37. Some research has shown that GO can stimulate the TLR4 (Toll-Like Receptor 4) on macrophages, which makes it possible to construct a vaccine adjuvant system 38. In our previous study, we designed a carnosine decorated-GO to deliver ovalbumin (OVA), which has good biocompatibility *in vitro*. The GOcar vaccination also induces a certain extant enhancement immune of the humoral and innate immune response 39. it is far from enough to fight against the SARS-CoV-2 pandemic, cancer, and other epidemics in the future.

PPR (pattern recognition receptors) agonists are wildly used to be a vaccine adjuvant in clinical and preclinical studies 40, 41. Especially, the TLR4 agonist Monophosphoryl Lipid A (MPLA) has been included in licensed vaccines for human papillomavirus and hepatitis B 35. TLR 9 (Toll-Like Receptor 4) agonist also attracted tremendous attention in SARS-CoV-2 vaccine development, caused by the ability to shift the T cell response to the Th1 response. There are at least four SARS-CoV-2 candidate vaccines that used TLR 9 agonists as an essential adjuvant 22, 42, 43. Additionally, CpG 1018 has been approved in the licensed vaccine Heplisav B 44.

To construct the SARS-CoV-2 vaccine formulation GCR which can improve the antigen-presenting function of Dendritic cell (DC) and elicit an Ag-specific adaptive immune response, we designed a GCP bi-adjuvant system to deliver SARS-CoV-2 spike RBD protein based on our previous research. The vaccine formulation consisted of carnosine-decorated graphene oxide (GOcar), CpG1018, and RBD protein, and the RBD sequence was acquired from Protein Data Bank 45.

The GCR vaccine has excellent RBD encapsulation and loading efficiency, which utilized less adjuvant to accomplish a higher concentration of rich immunogenicity RBD complex, and cut down the raw materials consumption in large-scale production, especially the expense of RBD recombinant protein manufacture. It also induced a massive cellular uptake and maturation of DC2.4 cells *in vitro*. The vaccine challenge experiment demonstrated that two dosages of GCR vaccine administration elicited a robust humoral immune response with enormous binding antibody responses. The RBD peptide pools assay showed the significant production of T helper cells (Th1) cytokines in CD4^+^ and CD8^+^ lymphocytes, recommending an antigen-specific T cells response. Furthermore, we prepared an MGCR microneedle patch vaccine to improve treatment compliance and vaccine stability, based on the GCR vaccine. The MGCR vaccine induced a similar binding antibody titer to the GCR vaccine, and the binding antibody titer also keep higher levels for more than 16 weeks in mice. The MGCR microneedle patch vaccine could be stored at room temperature for months. Meanwhile, the simple vaccination approach of microneedles allowed people to vaccinate by themselves at home in the epidemic area, avoiding the infected risk of aggregation. This study might offer a universal and effective vaccine adjuvant system to fight against the COVID-19 pandemic and other pandemics occurred in the future.

## Results and Discussion

### Preparation and characterization of GCR Nanovaccine

**Figure 1:**
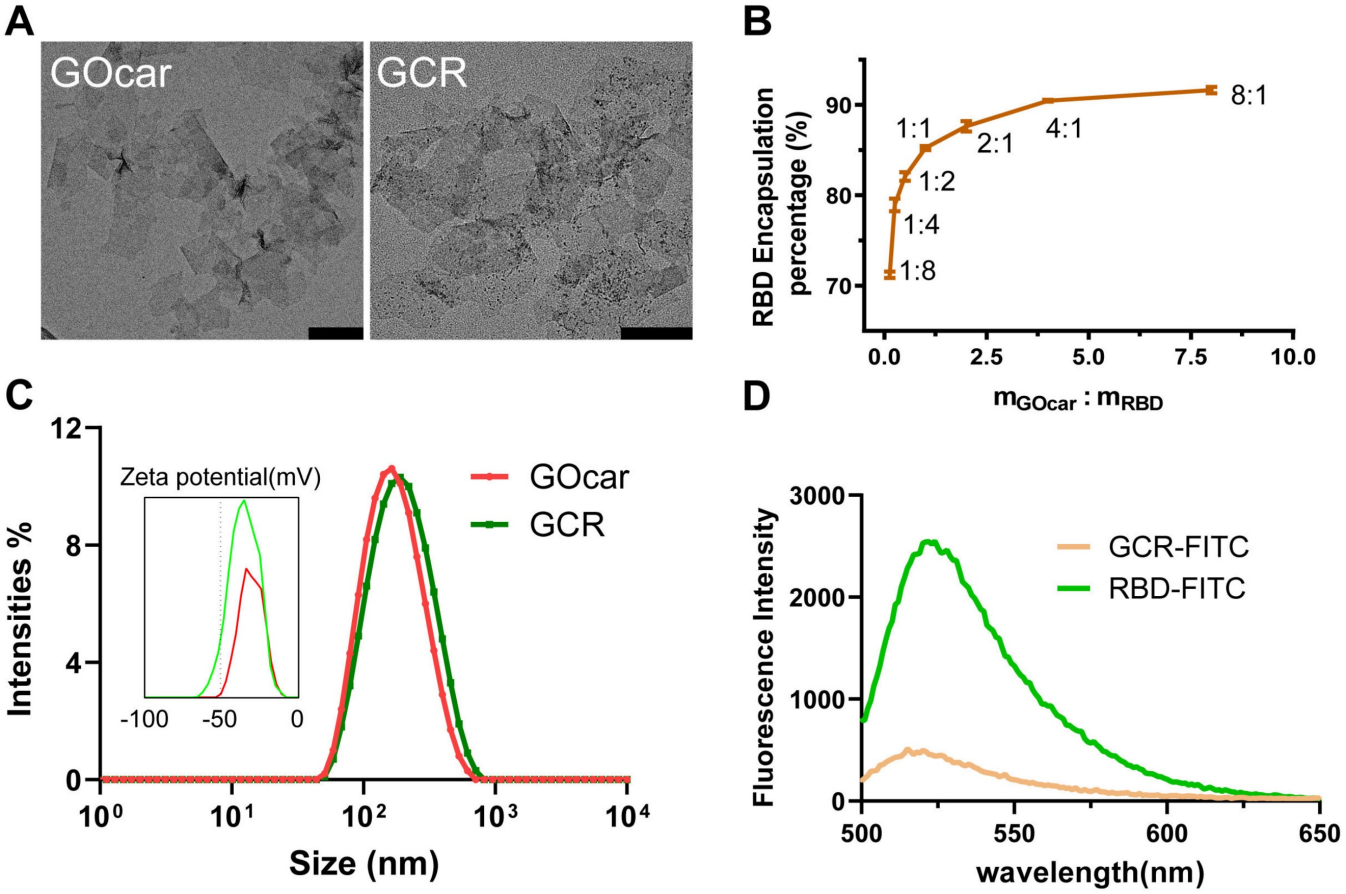
Chemo physical characterization of GCR vaccine. (A) The representative TEM image of GOcar and GCR (Scale bars = 200 nm). (B) Optimization of the GCR formulation by altering the binding ratio of RBD to GOcar (the mass ratio of the GOcar to RBD ranged from 1:8-8:1). (C)The hydrodynamic particle size and zeta potential distribution of GCR and GOcar. (D) The fluorescence emission spectrum of GCR-FITC and RBD-FITC with an equal RBD-FITC concentration. The excitation wavelength was 488 nm.

We synthesized GOcar following our previous work and attenuated it to 2mg/mL for the succeeding preparation of GCR. The amino acid sequence of RBD protein was shown in Figure S1. To investigate the antigen loading efficiency of GOcar, we labeled RBD with Fluorescein isothiocyanate to obtain FITC-decorated RBD (RBD-FITC), and set up a loading experiment with a range of the mass ratio of GOcar to RBD-FITC from 1:8 to 8:1. The Transmission Electron Microscope (TEM) images of GOcar and GCR vaccine was shown in Figure 1A. From the TEM image of GCR, we observed numerous dark points caused by RBD aggregations. The AFM images of GOcar and GCR also identified the RBD's successful loading in Figure S2. Due to the high protein absorption activity of GOcar, the RBD encapsulation efficiency was more than 70%, with an extremely low mass ratio of 1 to 8. As the mass ratio grew, the encapsulation efficiency increasing rate slowed down gradually, and reached a platform of 91% at 1 to 8 (Figure 1B). High concentration GO always leads to poor stability and flowability of mixture solution, so we chose a mass ratio of 2: 1 to prepare the GCR vaccine, the encapsulation efficiency and loading efficiency were nearly 89% and 30% respectively. As shown in Figure 1A and 1B, GCR and GOcar were similar in diameter of 130nm, and the RBD loading could slightly enhance the diameter of GOcar. The hydro-diameter also revealed a similar size of about 150 nm, and RBD loading caused more negative Zeta potential from -31mv to -34mv (Figure 1C). We also analyzed the fluorescence emission spectrums of FITC-decorated GCR (GCR-FITC) and RBD-FITC (Figure 1D). Free RBD-FITC has an obvious fluorescence emission peak at 522 nm wavelength, Once RBD-FITC was loaded in GOcar, the fluorescence intensity rapidly reduced by 82%, indicating the strong fluorescence quenching ability. We also investigated the RBD-releasing behaviors in different PBST buffers (pH 4.0 and 7.2). As shown in Figure S3. After 48 h incubation at 37°C, less than 20% RBD is released in pH 7.2 PBST buffer, and more than 70% RBD is released in pH 4.0 PBST buffer.

### Vaccine formulations induced cellular uptake and DC2.4 maturation

**Figure 2:**
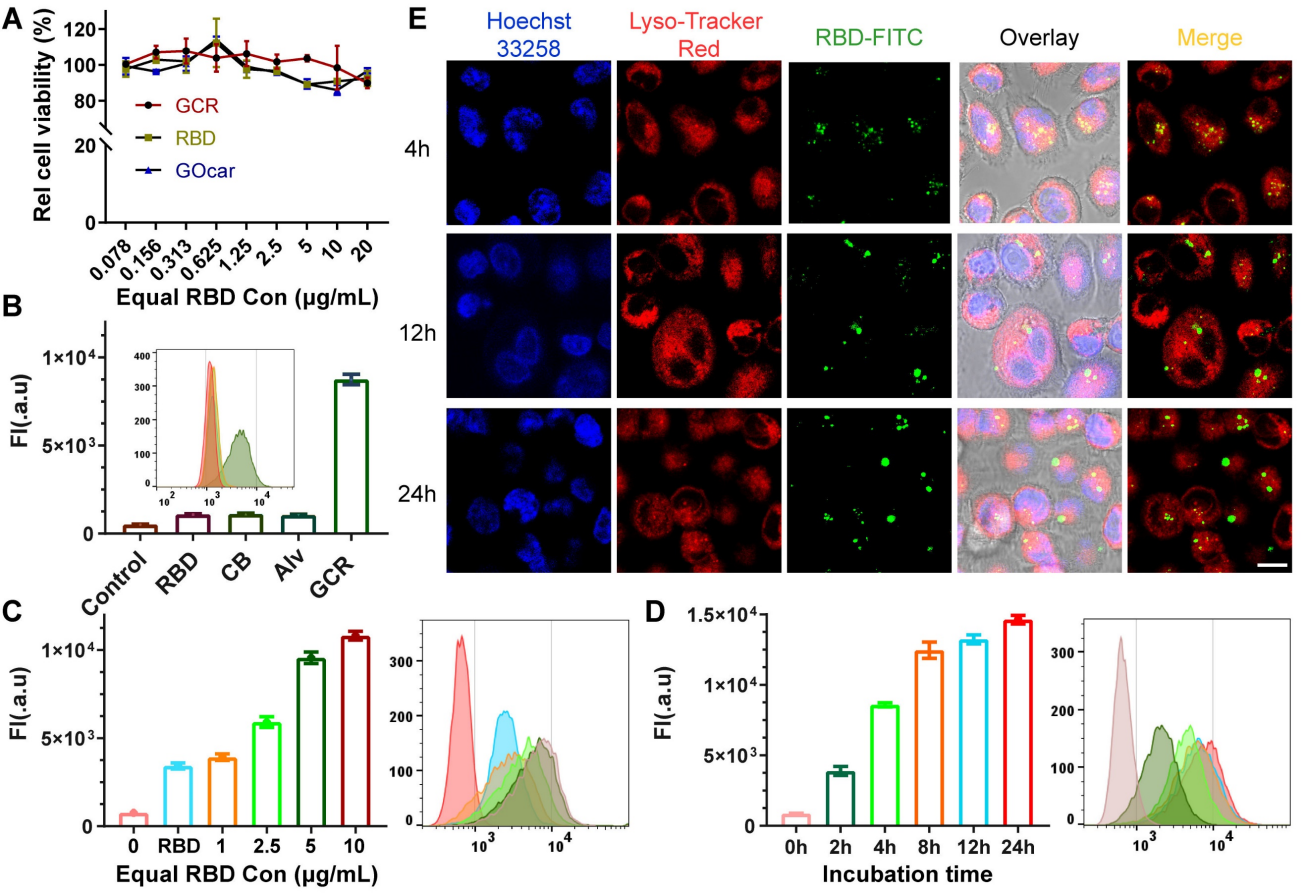
Intracellular uptake and colocalization of GCR-FITC *in vitro*. (A) Cell viability of DC2.4 cells after 24 hours incubation with RBD, GO-car, and GCR. All these formulations have equal RBD or GO-car concentration, and 1μg/mL of RBD was equivalent to 2μg/mL of GO-car and 3.3μg/mL of GCR. (B) DC2.4 cells intracellular uptake of different vaccine formulations with an equal RBD concentration of 5ug/mL after 24 hours incubation. (C) DC2.4 cells intracellular uptake of GCR with different GCR concentrations. The RBD concentration in the RBD group was 10 ug/mL. (D) cellular uptake of GCR by DC2.4 cells with a range of incubation times. The equal RBD concentration was 10 ug/mL. (E) CLSM of DC2.4 cells after incubation with GCR for 4, 12, and 24 hours and the Pearson's R values were 0.36, 0.12, and 0.13 respectively (Scale bar = 25μm). Pearson's R value was calculated by Colorc 2 plugin in Image J.

It was thought that a high concentration of graphene oxide would lead to immune toxicity in zebrafish 46. Hence, DC 2.4 cells were used to assess the cytotoxicity and cellular uptake of GCR (Figure 2A). The Cell Counting Kit (CCK-8) assay illustrated that either GOcar or GCR had negligible cytotoxicity in DC 2.4 cells at the maximum concentration of 20 μg/mL *in vitro*. To assess the cellular uptake behavior of GCR, the RBD-FITC was used as a model protein to prepare all the vaccine formulations. As shown in Figure 2B, the fluorescence intensity of free RBD groups increased 1-fold more than the negative control. The poor cellular internalized ability by lymphocytes also explained their poor immunogenicity. Meanwhile, due to the large particle size and poor stability of Imject alum, Alv (Imject alum vaccine) showed a weak cellular internalized quantity like free RBD. In comparison, the GCR vaccine led to an 8.1-fold increase in cellular uptake than free RBD and other formulations, which revealed its potent antigen delivery ability. The concentration-dependent experiments confirmed again in Figure 2C, that the uptake quantity of 1μg/mL GCR was 1.14-fold more than 10μg/mL free RBD. When equal RBD concentration reached 5μg/mL, cellular uptake quantity increased slowed down. Subsequently, we designed a time-dependent experiment to find out the optimum incubation time of the GCR vaccine (Figure 2D). The time-dependent experiment indicated that a 12-hour co-incubation was enough for DC 2.4 cellular uptake. In addition, the Confocal Laser Scanning Microscopic (CLSM) analysis demonstrated the colocalization of the GCR vaccine with the lysosome at 4 hours and separated at 12 hours and 24 hours (Figure 2E). Meanwhile, we used the Colorc 2 plugin of ImageJ to calculate Pearson's R value. And the Pearson's R values in different incubation groups (4, 12, and 24 hours) were 0.36, 0.12, and 0.13 respectively, the decreased Pearson's R values showed the reduced colocalization coefficient. As shown in Figure 1D, the GOcar could quench the fluorescence of RBD-FITC, and the fluorescence recovery and lysosome colocalized indicated the disaggregation of GCR in the lysosome. Furthermore, the separated lysosome localization revealed that the lysosome might have processed the RBD-FITC and released antigen peptides for following cross-presentation, which was essential for TCR recognition and cellular immune response stimulation.

**Figure 3:**
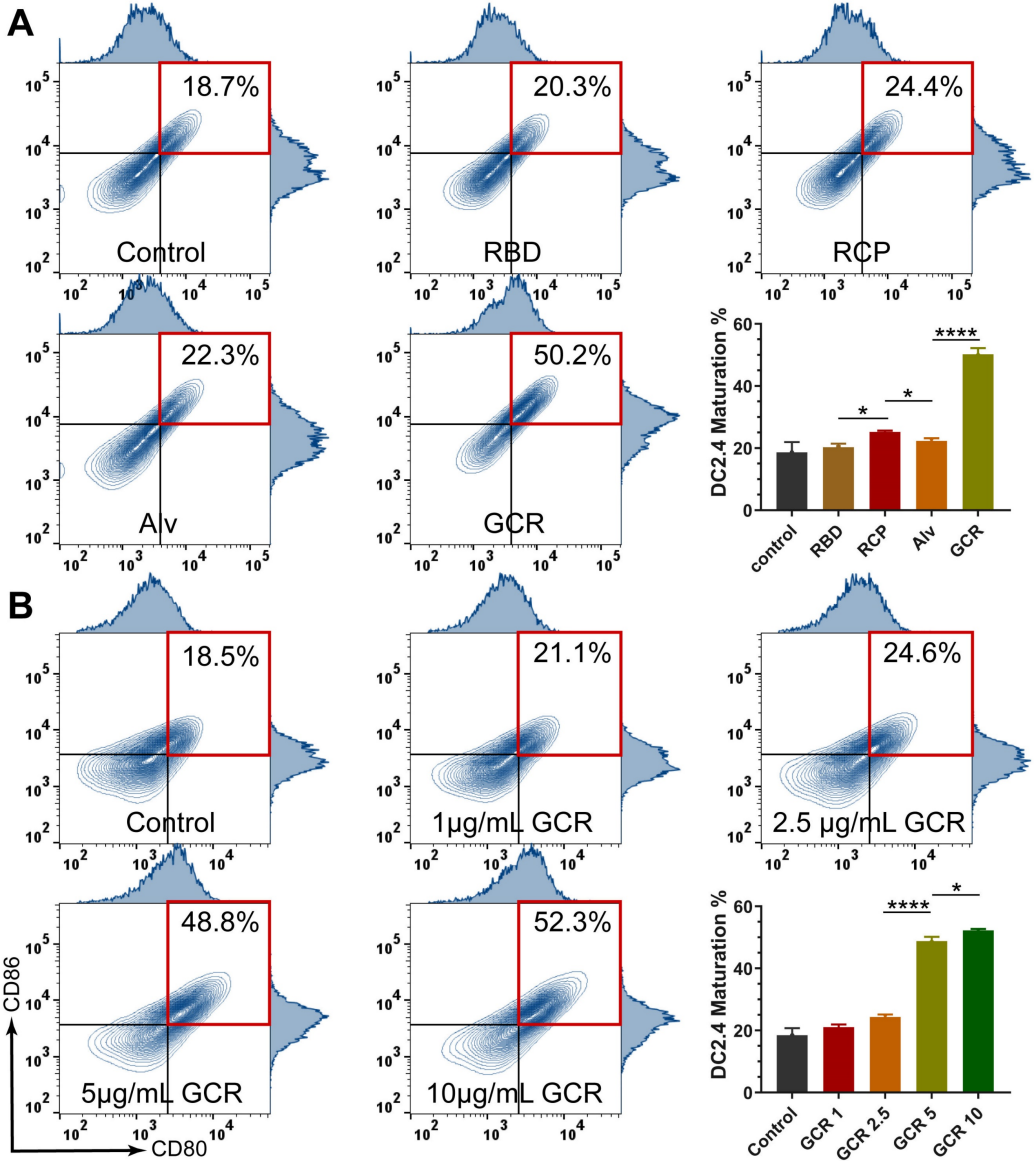
Vaccine formulations induced DC activation. (A) the representative flow cytometry spectrum of DC 2.4 cells maturation and quantitative statistics of DC 2.4 cells maturation were analyzed by flow cytometry after incubated with RBD, RCP, Alv, and GCR vaccines for 24 hours (all the formulations have equal RBD concentration of 5ug/mL). (B) the representative flow cytometry spectrum of DC 2.4 cells maturation and quantitative statistics of DC 2.4 cells maturation, were analyzed after incubation with different GCR concentrations for 24 hours. (* p < 0.05, ** p < 0.01, *** p < 0.001, **** p < 0.0001)

As we know, naive DCs could capture and process antigens, but only maturated DCs could present captured antigens to T cells and stimulate cellular immunity. Therefore, it is necessary to access the DC maturation by GCR vaccine induced. The Fluorescence-activated cell sorting (FACS) experiments illustrated that GCR stimulated a significant DC2.4 maturation rate of 50.2% after 24 hours of incubation; RBD and CpG mixture (RCP) induced a mildly increasing DC2.4 maturation rate; Other vaccine formulations had no contribution to improving DC maturation. (Figures 3A). CpG 1018 was one of the agonists of TLR9, the activation of TLR9 led to the increase of DC2.4 maturation in the RCP group. GCR vaccine could activate both TLR4 and TLR9 pathways leading to a higher DC2.4 maturation rate, meanwhile, the nuclein loading capability of GCR could induce a stronger activation than free CpG 1018. With the GCR concentration grown, the DC maturation reached a platform of about 50% at the concentration of 5μg/mL, which elicited a 2-fold of DC maturation rate than GCR 2.5, indicating the importance of adequate adjuvant concentration (Figure 3B).

### RBD-specific antibody response and pseudovirus neutralization ability

**Figure 4:**
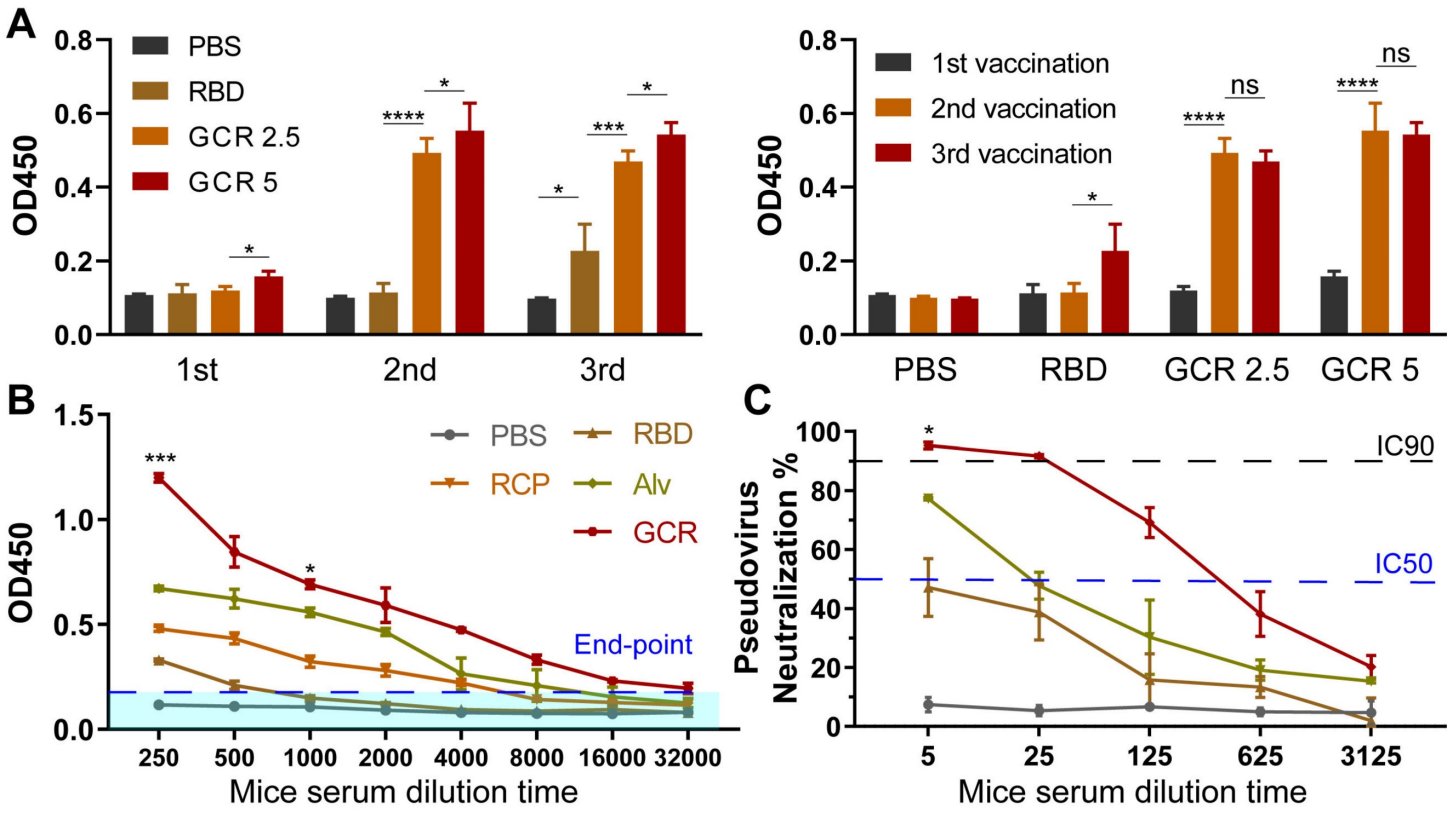
Vaccine-induced RBD binding IgG titers and neutralization antibody response. (A) Relative RBD binding IgG concentrations in mice serum after vaccinations. The mice serum was diluted 1000-fold for ELISA assays. (B) Binding IgG titers after 2^nd^ vaccination in subcutaneous, all the formulations have an equal RBD concentration of 5 μg/mL. 2-fold of the PBS group average OD450 value was set as the endpoint, and we calculated the end-point titers of vaccines respectively (RBD:770, RCP:5850, Alv:12213, GCR: 38380). (C) Pseudovirus neutralization activity of mice serum after 2^nd^ vaccination. The IC 90 and IC 50 of GCR were 30 and 312. The IC 50 of Alv was 23 (* p < 0.05, ** p < 0.01, *** p < 0.001, **** p < 0.0001)

To optimize the GCR vaccination dosage and frequency, we designed two-factor experiments. As shown in Figure 4A, a single vaccination of the majority vaccine formulations elicited negligible RBD binding response except GCR 5 which elicited a 1.5-fold OD450 value. With a boost of the 2^nd^ vaccination, both GCR 5 and GCR 2.5 induced a remarkable uplift of the IgG lever. The OD value of GCR 2.5 was increased about 3.5-fold than free RBD, as for GCR 5 was 4-fold. After the 3^rd^ vaccination, RBD finally elicited an RBD-binding antibody response, revealing the extremely low immunogenicity again. The antibody level of GCR 5 and GCR 2.5 had no obvious improvement, and GCR 5 also had a 0.1-fold higher OD value than GCR 2.5. These results implied that the bi-adjuvant system could extremely improve the immunogenicity of free RBD, and two dosages of administration of the GCR 5 vaccine were enough to elicit a higher binding antibody level. Subsequently, RBD-specific IgG titers of vaccine formulations were assessed with a stander Enzyme-linked immunosorbent assay (ELISA) experiment (Figure 4B). Both Alv and GCR vaccines induced adequate RBD-specific IgG titers with two dosages vaccination, and the end-point titer of GCR and Alv were increased 5.56-fold and 1.08-fold than the RCP group respectively. Meanwhile, we also accessed the pseudovirus neutralization capability of all the vaccine formulations in Figure 4C. The GCR vaccine not only displayed an extreme promotion of 2.14-fold IgG titer than Alv, but it also induced a stronger pseudovirus neutralization titer of about 13.6-fold of IC50 than Alv. Furthermore, we also monitored the weight change of all groups, the mice body weight of the experimental groups had negligible change after two dosage vaccination to the control group (Figure S4).

### GCR induced strong RBD-specific Type 1 Cellular response

**Figure 5:**
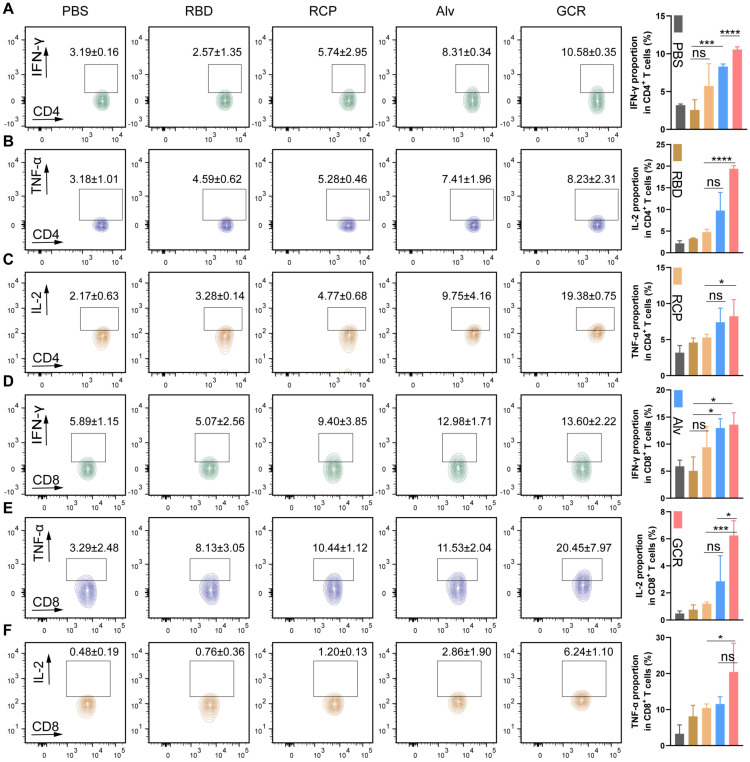
Vaccine-induced RBD-specific Type 1 cellular response. Balb/c was administrated in two doses of GCR vaccine formulations. After two weeks, the spleen lymphocytes were harvested to stimulate with RBD peptide pools and analyze with FACS multicolor staining assays. the representative flow cytometry spectrum and quantitative statistics of type 1 cellular cytokines expression. (A) IFN-γ^+^ CD4^+^ T cells; (B) TNF-α^+^ CD4^+^ T cells; (C) IL-2^+^ CD4^+^ T cells; (D) IFN-γ^+^ CD8^+^ T cells; (E) TNF-α^+^ CD8^+^ T cells; (f) IL-2^+^ CD8^+^ T cells. (ns p ≥ 0.05, * p < 0.05, *** p < 0.001, **** p < 0.0001)

To detect the production of the Th1 cytokines after vaccination, Balb/c mice were treated with vaccine formulations I.H. two times, including PBS, free RBD, RCP, Alv, and GCR (each vaccine formulation contains 5ug RBD and 1.5μg CpG 1018). After 2 weeks, the RBD-specific CD4^+^ and CD8^+^ T cells response induced by 2ug/mL RBD peptide pools were analyzed with multicolor staining FACS (Figure 5). We evaluated 3 kinds of Type 1 intracellular cytokines production in CD 4^+^ and CD8^+^ T cells, and the the gating strategy was shown in Figure S5A. Both GCR and Alv vaccine formulations enhanced the IL-2, IFN-γ, and TNF-α cytokines secretion in CD8^+^ and CD4^+^ T cells. Especially, the IL-2 proportion of GCR was increased by 0.98-fold and 1.20-fold than Alv in CD4^+^ and CD8^+^ T cells. (Figure 5C and 5F); the IFN-γ proportion of GCR was increased 0.27-fold than Alv in CD4^+^ T cells (Figure 5A); the TNF-α proportion of GCR was increased 0.77-fold than Alv in CD8^+^ T cells (Figure 5E). As we know, Alum could elicit humoral immune responses merely, but the accessory effect of CpG 1018 gave Alv the ability to elicit a degree of Th1 response. This phenomenon also explained the higher Th1 cytokine expression in the GCR group than Alv, the activation of both TLR 4 and TLR 9 of the GO adjuvant system extremely improves the Th1 mediating ability of GCR. Type 1 cellular cytokines concentration in spleen lymphocytes culture supernatant also significantly increased after 24 h incubation with RBD peptide pools (Figure S6).

### MGCR microneedle patch vaccine elicited RBD-specific antibody response

SC (Subcutaneous) injection should reach the hypodermis of the skin which allow large-volume solution (more than 1mL) delivery. But Compared to the dermis, hypodermis has poorly skin-resident APCs and lymphatic vessels. Subcutaneous injection could not directly involve the skin-resident APCs, only recruit leukocytes by inflammatory molecules secreted at the vaccination site. ID (Intradermal) injection is used to deliver antigen to the dermis, has a shallower injection position, and has less injection volume (about 0.1-0.2mL). However, there is a generous nerve ending in the dermis, ID would cause acute pain at injection sites, and it also requires skilled medical personnel. Hence, we developed a microneedle delivery system MGCR based on the GCR vaccine to promote vaccination compliance. the excellent stability at ordinary temperatures has proven the possibility of a new vaccination approach to cut down the budget for vaccination against SARS-Cov-2 and future pandemics 47.

**Figure 6:**
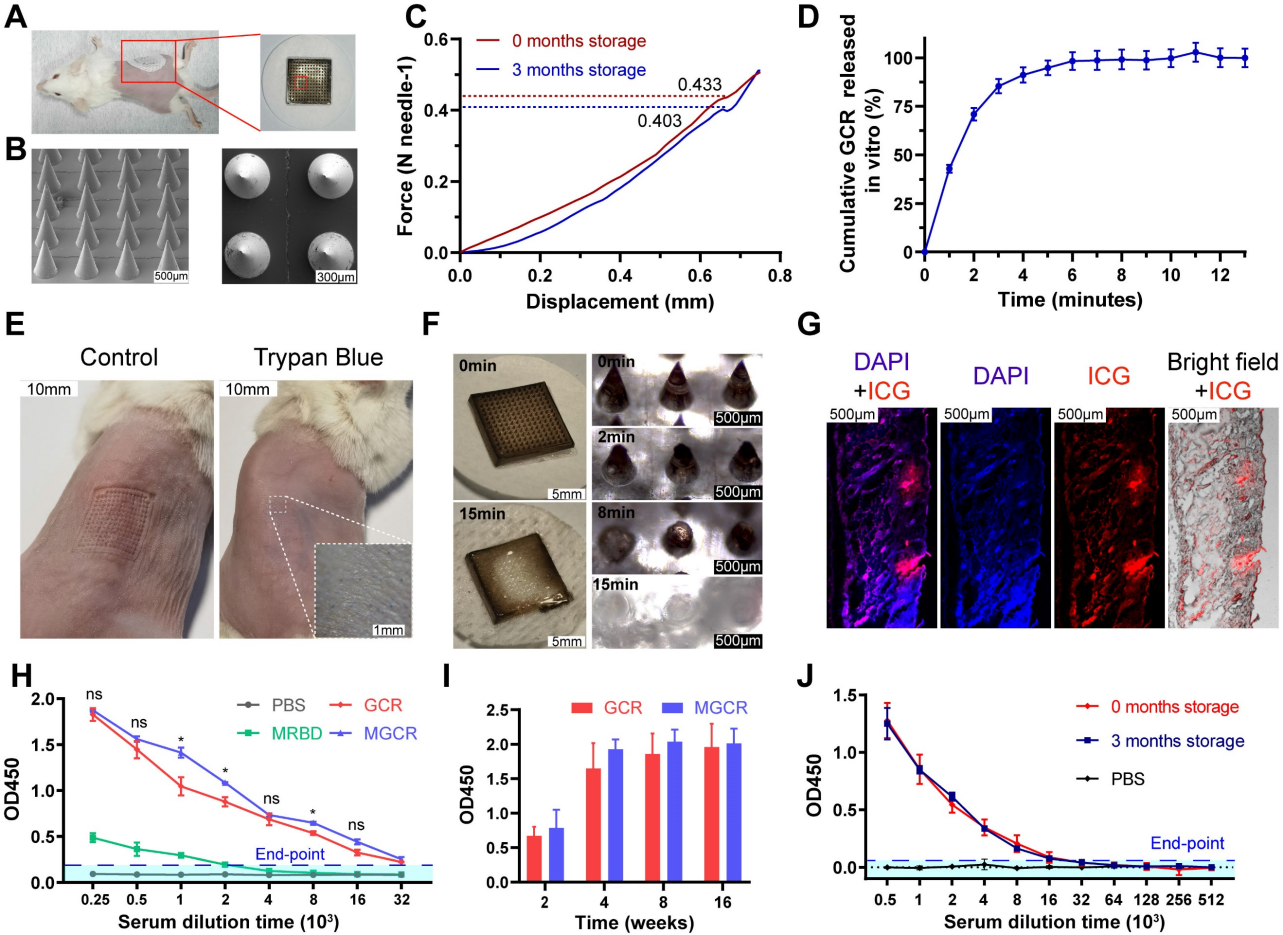
Physical characterization and neutralization antibody titers of MGCR microneedle patch vaccine. (A) Representative photograph and vaccination site of the microneedle. (B) The front and left view of MGCR microneedles with SEM imaging. (C) Mechanical behavior of MGCR with different storage times under compression administered by a vertical force. (D) Cumulative GCR release *in vitro* from MGCR microneedle patches in PBS solution at 37°C. (E) Mouse skin penetration experiment: skin pinhole (left) and skin pinhole stained with 0.4% trypan blue (right) after MGCR was administrated for 30 seconds. (F) Representative microneedle photographs (left) and microscopy images (right) after application to mouse skin for a different duration. (G) Histological images of MGCR applicated to mouse skin. (H) RBD specific binding antibody titers of vaccine formulations after 2^nd^ vaccination. 2-fold of the PBS group average OD450 value was set as the endpoint, and we calculated the end-point titers of vaccines respectively (MRBD:3145, GCR: 81594, MGCR: 150124). (I) ELISA analysis of sustained binding antibody concentration at 4, 8, and 16 weeks after 2^nd^ vaccination. (J) RBD-specific binding antibody titers of MGCR microneedles with 3 months storage at RT. All the mice were vaccinated at weeks 0 and 2^nd^ respectively. (* p < 0.05, ** p < 0.01).

Hyaluronic acid and sucrose were used as an excipient and stabilizers of the MGCR microneedle patch vaccine respectively 48. Each MGCR patch contained 5.4μL GCR vaccine formulation (10μg GO-car, 5μg RBD, and 1.5μg CpG 1018), and was injected into the right stomach of mice (Figure 6A). The optical photograph in Figure 6A demonstrated that every patch comprised a 13*13 microneedles array. The scanning electron microscope image of MGCR revealed the fine structure at different views (Figure 6B). Each microneedle was designed as a stander cone. The microneedle length was measured as 750μm which was long enough to penetrate the dermis layer. To illustrate the skin penetration properties of MGCR microneedles, we measured the mechanical strength of MGCR microneedles. As shown in Figure 6C, each microneedle could tolerate compressive forces of ≥0.4 N, which is expected to enable skin puncture. Meantime, we investigated the microneedle penetration ability on mouse skin. The MGCR microneedle patch was detached after application for 30 seconds, the clearly pinholes array were observed and healed in 15 minutes (Figure S7).Subsequently, the 0.4% trypan blue was used to stain punctured mouse skin. As shown in Figure 6E, clearly blue skin pinhole could be observed in an optical microscope, which proved the skin penetration ability of MGCR microneedles. The Hyaluronic acid (HA) based microneedle could be dissolved within 15 minutes after administrated (Figure 6D and 6F). Furthermore, skin histological sections in microneedle application sites were used to explore the penetration properties of microneedles (Figure 6G). The red fluorescence distributed into the whole skin, which indicated the microneedles can completely penetrate the mouse epidermis enter the dermis, and dissolve in the dermis, then acted as the GCR vaccine. We also investigated the distribution of the MGCR in Balb/c mice (Figure S8). GCR and MGCR) significantly reduced liver/kidney accumulation and all the inguinal lymph nodes nearby injected sites showed obvious fluorescence intensity 24 hours after the vaccines were administrated.

Subsequently, we compared the RBD-specific binding antibody titers between GCR and MGCR vaccines. MGCR had a similar end-point titer to the GCR vaccine in Figure 6H. The sustained RBD-specific binding antibody in mouse serum was also monitored for 16 weeks. Both GCR and MGCR groups maintained a significant binding antibody level after two dosage vaccination (Figure 6I), which could provide long-term protection against SARS-Cov-2 virus infection. Meanwhile, we also explored the long-term stability of MGCR. After 3 months of storage at RT, the mechanical strength of the MGCR microneedles have no significant change (Figure 6C). In the mouse immunization experiment, MGCR microneedles stored 3 months RT still showed similarly remarkable RBD-specific IgG levels compared to the fresh preparation MGCR microneedle (Figure 6J).

**Figure 7:**
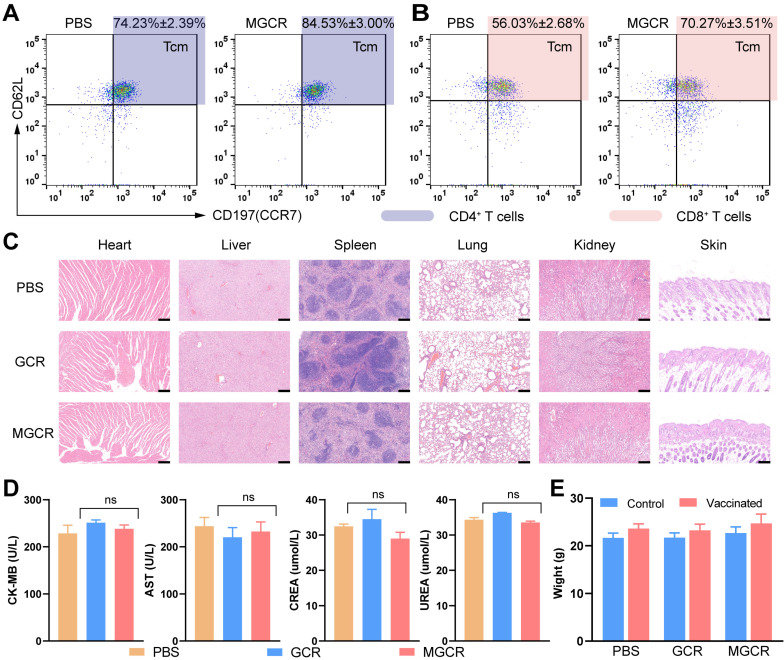
Immunological memory and biocompatibility of MGCR microneedle patch vaccine. (A) Flow cytometry analysis of CD4^+^ T_cm_ cells (gated on CD45^+^CD3^+^CD4^+^) and (B) CD8^+^ T_cm_ cells proportion (gated on CD45^+^CD3^+^CD8^+^) in mice spleen with two doses of MGCR microneedles administrated. After two weeks, the spleen lymphocytes were harvested and analyzed with FACS multicolor staining assays (C) hematoxylin-eosin staining of major tissue slices (heart, lung, spleen, liver, kidney, and skin) at week 2 after 2^nd^ vaccination. (Scale bar= 200μm) (D) Serum biochemistry markers (including CK-MB, AST, CREA, UREA). (e) Mice' weight changes after 2^nd^ vaccination.

T_cm_ (Central Memory T cell) has long-term memory effects after inoculation. Once antigen rechallenge, T_cm_ could rapidly proliferate and differentiate into effective memory T cells to protect against virus infection. To explore memory immune responses induced by MGCR, we analyzed the central memory T cell (Tcm) proportion in splenocytes with the gating strategy in Figure S5B, the representative flow cytometry spectrum and quantitative statistics were displayed in Figure 7A and Figure 7B. Compared to the PBS group, the MGCR microneedle could significantly raise the Tcm proportion in the mouse spleen. Meantime, we detected the MGCR-generated RBD-specific Type 1 cellular response and revealed the adequate Th 1 cytokines expression of MGCR, which was equivalent to GCR vaccine-induced (Figure S9).

To assess the safety of MGCR and GCR vaccine formulations *in vivo*, we harvested major tissues (heart, lung, spleen, liver, kidney, and injection sites skin) at week 2 after 2nd vaccination, and stained them with hematoxylin-eosin kits. The Hematoxylin-Eosin (HE) Stain revealed no obvious organ damage in both GCR or MGCR vaccine formulation groups (Figure 7C). Furthermore, blood biochemistry makers were used to evaluate cardiac, liver, and kidney damage (Figure 7D). Compared with the PBS group, the CK-MB & AST (markers of cardiac damage) and CREA & UREA (markers of kidney damage) levels have no statistically significant alteration (T.TEST analysis, P≥0.05). In addition, we monitored the body changes at the beginning and end of the mouse vaccination experiment, as shown in Figure 7E, the mice body weight of the experimental groups had negligible change after two dosages of vaccination than the control group.

## Conclusion

In summary, we designed a CpG 1018 and graphene oxide-based bi-adjuvant system to deliver SARS-CoV-2 RBD protein and developed a novel subunit recombinant protein vaccine of GCR against COVID-19 pandemics. Due to the efficient antigen absorption ability of graphene oxide, GCR obtained excellent antigen loading efficiency and encapsulation efficiency. Additionally, the cellular uptake experiment also demonstrated that the GCR vaccine could mediate efficient cellular uptake in DC 2.4 cells. Subsequently, a laser confocal image revealed that RBD has released from the lysosome into the cytoplasm, which was essential for antigen cross-presentation and T-cell response stimulation. In mouse vaccination research, The GCR vaccine could elicit stronger RBD-specific and pseudovirus neutralization antibody response than the conventional alum adjuvant system after two dosages of I.H. The spleen lymphocytes analysis also indicated that the GCR vaccine elicited robust RBD-specific Type 1 Cellular response, and induced strong CD8^+^ cells response. Based on the GCR vaccine, we prepared an MGCR microneedle vaccine to enhance vaccine stability. MGCR microneedles could easily penetrate mouse skin, rapidly dissolve, and release the GCR vaccine. The HE staining and quick skin recovery at administrated sites revealed excellent biocompatibility of MGCR microneedles. The MGCR microneedles significantly elevated the Tcm proportion in splenocytes and induced a sustained high level of RBD-specific binding antibody concertation more than 16 weeks after two dosage vaccinations, which stimulated a similar RBD-specific antibody response to the GCR vaccine. The MGCR microneedle patch vaccine also could be stably stored at room temperature for several months and administrated without medical staff, which maximizes the vaccine distribution efficiency.

Compared with some traditional vaccine adjuvants, the MGCR microneedle patch vaccine has shown several unique advantages. At first, the superb antigen absorption ability tremendously decreased the usage of antigens and adjuvants. Less usage of adjuvants would lead to fewer side effects caused by adjuvants and greatly improve vaccine safety. Secondly, most of the approved vaccines need strict transportation conditions, for example, the nuclein vaccine must be stored in ultra-cold storage of -20°C, and other vaccines also need 4-8°C storage temperature. The MGCR microneedles patch could be stored at room temperature for 3 months. Meanwhile, the patch was designed convenient enough for people to handle and vaccinate at home in an epidemic area, which could avoid the spread of risk by people's aggregation of vaccination. All these advantages could maximally decrease the manufacture and distribution cost, and maximize the vaccination coverage in poor medical recourse areas.

## Methods

### Materials

RBD was purchased from Sanyou Biopharmaceuticals Co., Ltd (Shanghai, China). CpG 1018 was purchased from GenScript Co., Ltd (Nanjing, China). Carnosine, Hyaluronic acid, and sucrose were purchased from Sigma-Aldrich (Shanghai, China). 5/6-FAM SE, cy5-nhs ester and Imject Alum was purchased from ThermoFisher Scientific. CCK-8 kits were purchased from Yeasen Biotechnology Co., Ltd (Shanghai, China). Hoechst 33258, LysoTracker Red, TMB (3,3′,5,5′-Tetramethylbenzidine), PMA, Ionomycin, Brefeldin A were purchased from Beyotime Biotechnology Co., Ltd (Nantong China). FITC anti-mouse CD11c, PE anti-mouse CD80, APC anti-mouse CD86, Zombie NIR™ Fixable Viability Kit, PE/Cyanine7 anti-mouse CD3, FITC anti-mouse CD4, and PerCP/Cyanine5.5 anti-mouse CD8, Brilliant Violet 421™ anti-mouse IL-2, PE anti-mouse IFN-γ, Brilliant Violet 510™ anti-mouse CD45, APC-anti-CD197, PE-anti-CD62L, and APC anti-mouse TNF-α were purchased from BioLegend, Inc. HRP goat anti-mouse IgG, Anti-SARS-CoV-2 Spike Glycoprotein RBD antibody purchased from Abcam (shanghai). Pseudovirus and RBD peptide pools were purchased from Sino Biological. IFN-γ ELISA Kits, IL-2 ELISA Kits, and TNF-α ELISA Kits were purchased from PeproTech, Inc.

### Preparation of GCR vaccine

Graphene oxide was synthesized through a typical Hummers method, and suspended in ultrapure water at a concentration of 2 mg/mL. Then the Go was modified with carnosine to obtain GOcar according to our previous studies. 9 mL GO was mixed with 1mL 10-fold phosphate buffer (pH=6.0) and ultrasonicated 10 min at 4°C. 4.72 mg of EDC and 5.35 mg of Sulfo-NHS were added to GO aqueous solution under ultrasonicated. The mixture was gently stirred for 15min to activate the carboxyl group at 25°C. After removing the excessive EDC/Sulfo-NHS from the reaction solution by ultrafiltration, 18 mg of carnosine was added to the above mixture and stirred for 4 hours at RT. The crude product solution was purified by dialyzing against ultrapure water for 24 hours. Finally, the pure GOcar was concentrated to a certain concentration of 2 mg/mL and stored at 4°C 39.

To prepare the GCR vaccine, GOcar, RBD, and CpG were dispersed in PBS with a mass ratio of 20:10:3. The mixture was mildly vortexed for 30min, and stored at 4°C.

### RBD loading efficiency of GCR vaccine

The loading efficiency of GCR was confirmed by FITC-labeled protein. The RBD was decorated with 5/6-FAM SE for RBD microfluorometric determination, and the fluorescence spectrum of RBD-FITC and GCR-FITC was detected with a Fluorescence Spectrometer.

The mass ratio of GOcar to FITC-RBD ranged from 0.125:1 to 1:0.125. At first, FITC-RBD was incubated with GOcar for 30min at RT. The mixture solution was centrifuged at 10,000 rpm for 30 min to obtain supernatant, and the fluorescence intensity of the free FITC-RBD in the supernatant was measured by an automatic microplate reader (PerkinElmer Enspire) to conform to the loading efficiency and encapsulation efficiency of RBD.

RBD encapsulation efficiency = (total RBD- free RBD) / total RBD

RBD loading efficiency = (total RBD- free RBD)/ total formulation

### Cytotoxicity of GCR vaccine

To evaluate the cytotoxicity of materials and GCR, DC2.4 cells were incubated in 96 well plates at the density of 3×10^3^ cells per well for 24 h. After that, the sample was treated with GCR and materials for 48 h at equal concentrations of RBD (0.078 μg/mL to 20 μg/mL) or GOcar (0.156 μg/mL to 40 μg/mL). CCK-8 assay was used to reveal the cell cytotoxicity of materials and GCR.

### Cellular uptake of the GCR vaccine

To investigate the difference in cellular uptake ability of GCR and other vaccine formulations. DC2.4 cells were incubated in 24 well plates at the density of 3×10^4^ cells per well for 24 hours. The DC2.4 cells were treated with GCR and other formulations at an equal RBD-FITC concentration of 5 μg/mL for 24 h. Then we harvested the samples and washed them 3 times with PBS at 4°C, after that the cell samples were suspended with 4°C PBS for FACS analysis (CytoFLEX, BECKMAN).

To investigate the time-dependent and concentration-dependent of cellular uptake of GCR. DC2.4 cells were incubated in 24 well plates at the density of 3×10^4^ cells per well for 24 h. The DC2.4 cells were treated with a series of GCR-FITC (from 1μg/mL to 10μg/mL) and incubated for 1 to 24 hours. Then we harvested the samples with 4°C PBS washed them 3 times, and suspended them with 4°C PBS for FACS analysis.

To investigate the intracellular distribution of GCR, DC2.4 cells were seeded on coverslip placed in 24 well plates at a density of 3×10^4^ cells per well and incubated for 24 h. The DC2.4 cells were treated with an equal RBD-FITC concentration of 5 μg/mL and incubated for from 4 to 24 h. The cells were treated with Hoechst 33258 and LysoTracker Red before being harvested. The coverslips were fixed with 4% paraformaldehyde and imaged with CLSM (Leica TCS SP8 STED 3X).

### DC maturation of the GCR vaccine

To detect vaccine-induced DC maturation *in vitro*, DC2.4 cells were incubated in 24 well plates at the density of 3×10^4^ cells per well for 24 h. The DC2.4 cells were treated with a series of GCR (from 1μg/mL to 10μg/mL) and incubated for 1 to 24 hours. Then we harvested the samples with 4°C PBS washed them 3 times, and suspended them with 4°C PBS. Then cell samples were stained with FITC anti-mouse CD11c, PE anti-mouse CD80, and APC anti-mouse CD86, and analyzed by FACS.

### Mouse immunization and serum collection

The Balb/c mice were vaccinated 2 or 3 times by hypodermic injection with PBS, RBD vaccine (5ug RBD), RCP vaccine (5ug RBD and 1.5ug CpG1018), Alv vaccine (50ug alum adjuvant, 5ug RBD and 1.5ug CpG1018), GCR5 (10ug GOcar, 5ug RBD and 1.5ug CpG1018) and GCR2 (5ug GOcar, 2.5ug RBD and 1.5ug CpG1018). Each vaccine was injected at an interval of 2 weeks.

The Mouse Submaxillary vein blood was collected at 2 weeks post 2^nd^ or 3^rd^ vaccination and coagulated for 2 hours to obtain serum for IgG detection and neutralizing antibody response.

### Enzyme-linked immunosorbent assay

The IgG level of mouse serum after vaccination with various vaccine formulations was assessed by ELISA. To precoat RBD protein, RBD was diluted to a concentration of 3μg/mL with pH 9.0 CB (Carbonate-Bicarbonate Buffer) and incubated in 96-well Elisa plates overnight at 4°C, then blocked for 2 hours with casein blocking solution at 37°C. The mouse serum has diluted a series of times with 3% BSA solution and added to Elisa plates for 2 hours of incubation at 37°C. After that, the HRP goat anti-mouse IgG was added to bind to captured neutralizing antibody and co-incubated for 1h at 37°C. TMB solution was used to react with HRP enzyme and stopped with 1M sulfuric acid solution. The absorbance at 450 nm and 630 nm of the mixed solution was measured by an automatic microplate reader. A washing procedure was executed after each experimental operation with an automatic plate washer.

To investigate the time-dependent and dosage-dependent of the GCR vaccine, the mouse serum was diluted 1000-fold and assessed by ELISA.

### Pseudovirus neutralization assay

Diluted mouse serum was mixed with Pseudovirus with a volume ratio of 1:1 and incubated at 37°C for 1 hour. Subsequently, these mixtures were added in pre-plated ACE2-expressing 293T cells and co-incubated for 48 hours. DMEM culture was used to be a negative control and commercial antibody as a positive control (0.42mg/mL). Later, a cell-lying buffer was used to release luciferase, and luciferase substrate was added to detect the activity of pseudovirus. The relative bioluminescence intensity revealed the neutralization of mouse serum. The reed-Muench method was used to calculate the IC50 and IC90.

### Lymphocytes analysis

Flow cytometric multicolor assay was designed to analyze cellular immunity and vaccine-induced cytokine secretion of splenocytes. We harvested mice spleen and prepared to lymphocyte single-cell suspension with a stander Lymphocytes Isolation Assay after 2 weeks post 2nd vaccination. Each sample was planted in 24-cell plates with a cell density of 10 million per mL. The experimental group was stimulated with an RBD peptide pool (2μg/mL), and the control group was stimulated with DMSO and PMA. We harvested the supernatants to detect cytokine production with ELISA Kits.

Each sample was pretreatment with Ionomycin/Brefeldin A cocktail for 6 hours before cell staining. Then samples were stained with Zombie NIR™ Fixable Viability Kit to exclude dead cells. PE/Cyanine7 anti-mouse CD3, FITC anti-mouse CD4, and PerCP/Cyanine5.5 anti-mouse CD8 were used for cell surface staining. After that, each sample was permeabilized and stained with Brilliant Violet 421™ anti-mouse IL-2, PE anti-mouse IFN-γ, and APC anti-mouse TNF-α. Finally, all the samples were analyzed with FACS.

Flow cytometric multicolor assay was designed to analyze the memory T cells response of splenocytes. We harvested mice spleen and prepared to lymphocyte single-cell suspension with a stander Lymphocytes Isolation Assay after 2 weeks post 2nd vaccination. Then samples were stained with Zombie NIR™ Fixable Viability Kit to exclude dead cells. Brilliant Violet 510™ anti-mouse CD45, PE/Cyanine7 anti-mouse CD3, FITC anti-mouse CD4, PerCP/Cyanine5.5 anti-mouse CD8, APC-anti-CD197 and PE-anti-CD62L were used to identify the central memory T cell. Finally, all the samples were analyzed with FACS.

### Microneedles patch vaccines preparation

To prepare MGCR and RBD microneedle (MRBD), 30%(m/m) Hyaluronic acid and 10% (m/m) sucrose were added to the GCR vaccine formulation which was pre-concentrated to 1.21 mg/ml of RBD, the final RBD concentration of mixture formulation was 0.93mg/mL. Subsequently, the mixture formulation was slowly injected in a microneedle pattern after uniformed with mild stirring and centrifugated at 3000rpm for 5 minutes. The final product was dried at room temperature for 24 hours and packaged in a dry environment.

### Microneedles patch shin penetration and dissolution

To evaluate the penetration of the MGCR microneedle, we administrated the MGCR microneedle on mouse skin for 30 seconds, then remove the microneedle. Using a digital camera to observe the skin healing status every 3 minutes. Subsequently, 0.4% trypan blue was used to stain the pinhole.

Microneedles loaded with fluorescent dye (ICG) were administrated on the mouse skin for 15 minutes. The administrated site skins were examined by CLSM (Leica TCS SP8 STED 3X) to identify the MGCR microneedles embedded in the skin.

The MGCR microneedles were administrated on mouse skin for 0, 2, 8, and 15 minutes, then remove the microneedles and observe the needle dissolution status with upright Light Microscopes.

**GCR release from MGCR microneedles**

To investigate GCR release behaviors *in vitro*, MGCR was stuck on the wall of the 100 mL volume beaker. 50 ml PBS buffer was added to the beaker. The breaker was incubated in a water bath at 37 °C and stirred at 50 r.p.m. 1 ml release medium was collected and replaced with 1 mL fresh medium Each minute.

### Microneedle immunization and serum collection

The Balb/c mice were vaccinated 2 times by hypodermic injection with PBS, GCR vaccine, MGCR microneedle, and MRBD microneedle in the mouse's right stomach. Each vaccine was administrated at an interval of 2 weeks.

The Mouse Submaxillary vein blood was collected at 2 weeks post 1^st^ vaccination and coagulated for 2 hours to obtain serum for IgG detection and binding antibody response. Every blood collection has an interval of 2 weeks.

### IgG titer of MGCR microneedle patch vaccine

The MGCR IgG titer was detected with the above ELISA method. The sustained neutralization antibody also was detected by the ELISA method with the 1000-fold dilution mouse serum. To assess long-term immunogenicity, we evaluated the serum IgG titers at 0, 2, 4, 8, and 16 weeks.

### *In vivo* biodistribution

We used cy5-decorated RBD to illustrate the distribution in Balb/c mice. Prepared cy5- labeled vaccines were administrated. IVIS imaging system was used to investigate the organ distribution after 24 h.

## Supplementary Material

Supplementary figures and tables.

## Figures and Tables

**Scheme 1:**
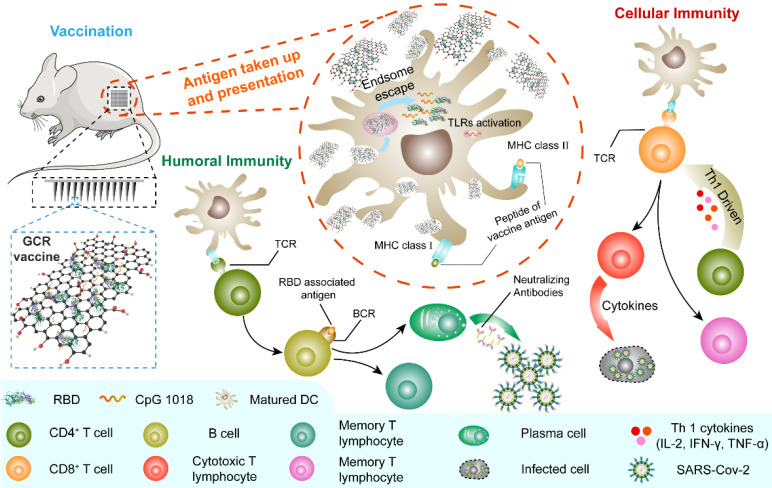
The generation of humoral and Th1-driven cellular immune response to MGCR microneedle patch (and GCR) vaccine. The MGCR was patched on the back of the mouse (GCR vaccine was injected I.H), and GCR was taken up by DCs. Subsequently, GCR was disaggregated in the endosome and released in the cytoplasm. TLR 9 and TLR 4 were activated by CpG 1018 and GOcar respectively, additionally inducing the DC maturation. Matured DC could process the RBD protein and activate T cells through the binding of MHC molecules of DC and T cell receptor (TCR), induced high-level RBD specific neutralization antibody for GCR vaccine to protect individuals, and elicited enormous Th 1 cellular response against SARS-CoV-2 infection in the future.

## Abbreviations

R&D): Research and development
RBD): Receptor-Binding Domain
GO): graphene oxide
GCR): graphene oxide-based complex adjuvant nanovaccine
MGCR): microneedle patch vaccine
Alv): Alum adjuvant vaccine
Ad): Adenovirus
CanSino Biological): Convidecia
GO): graphene oxide
Toll-Like Receptor 4): TLR4
Toll-Like Receptor 9): TLR9
OVA): ovalbumin
pattern recognition receptors): PPR
DC): Dendritic cell
GOcar): carnosine decorated graphene oxide
Th1): T helper cells
RBD-FITC): FITC-decorated RBD
TEM): Transmission Electron Microscope
GCR-FITC): FITC-decorated GCR
CCK-8): Cell Counting Kit
Imject alum vaccine): Alv
CLSM): Confocal Laser Scanning Microscopic
FACS): Fluorescence-activated cell sorting
RCP): RBD and CpG mixture
ELISA): Enzyme-linked immunosorbent assay
HE): Hematoxylin-Eosin
markers of cardiac damage): CK-MB & AST
markers of kidney damage): CREA & UREA
