# Supplementary material for: Developing an efficient MGCR microneedle nanovaccine patch for eliciting Th 1 cellular response against the SARS-CoV-2 infection
Source: Theranostics. 2023 Sep 4;13(14):4821–35. doi: 10.7150/thno.83390 (PMC10526668; doi:10.7150/thno.83390)

Supporting Information

Developing an efficient MGCR microneedle nanovaccine patch for eliciting Th 1 Cellular response against the SARS-CoV-2 infection

Ang Gao[1,2], Yunsheng Chen[1,3], Hui Liang[2], Xinyuan Cui[3], Amin Zhang[1,2], Daxiang Cui[1,2]*

[1]Institute of Nano Biomedicine and Engineering, Shanghai Engineering Research Center for Intelligent Instrument for Diagnosis and Therapy, School of Sensing Science and Engineering, Shanghai Jiao Tong University, Shanghai 200240, PR China
[2] National Engineering Research Center for Nanotechnology, 28 East Jiangchuan Road, Shanghai 200241, China.
[3] Radiology Department of Ruijin Hospital, Shanghai Jiao Tong University School of Medicine, 197 Ruijin Second Road, Shanghai 200025, China

*Corresponding author.
E-mail address:
dxcui@sjtu.edu.cn (D. Cui)

Figure S1. The detailed amino acid sequence of RBD protein.

RVQPTESIVRFPNITNLCPFGEVFNATRFASVYAWNRKRISNCVADYSVLYNSA
SFSTFKCYGVSPTKLNDLCFTNVYADSFVIRGDEVRQIAPGQTGKIADYNYKL
PDDFTGCVIAWNSNNLDSKVGGNYNYLYRLFRKSNLKPFERDISTEIYQAGST
PCNGVEGFNCYFPLQSYGFQPTNGVGYQPYRVVVLSFELLHAPATVCGPKKS
TNLVKNKCVNFHHHHHH

Figure S2. (A) Tapping mode AFM images of GOcar (up) and GCR (bottom). (B) Height profile of GOcar and GCR.

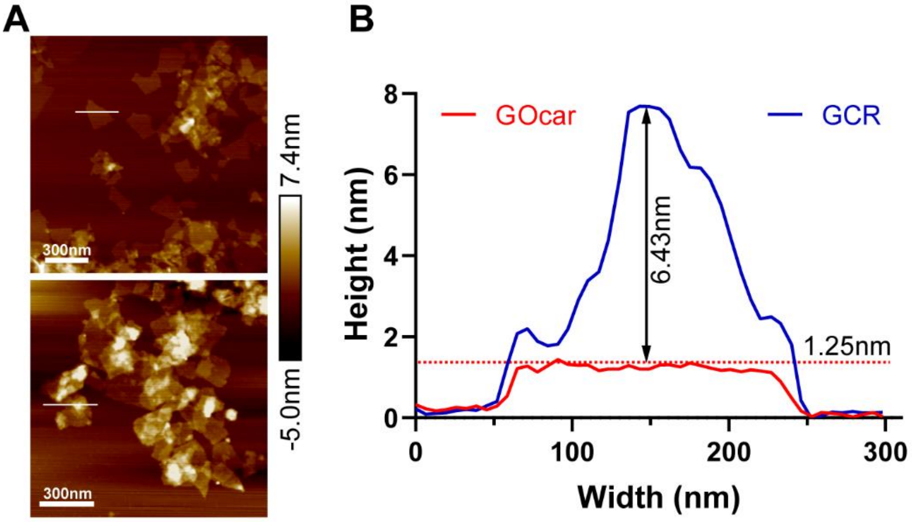

Figure S3. In vitro cumulative RBD release in PBST solution at 37℃

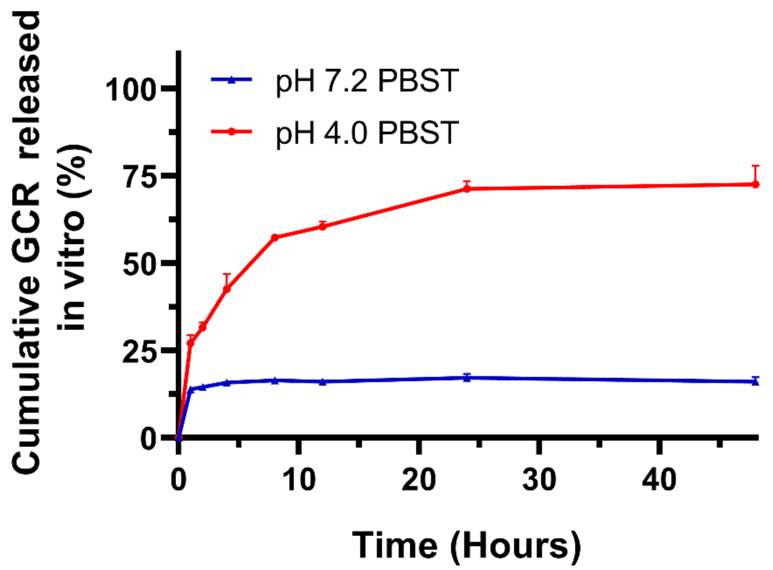

Figure S4. Mice weight changes after 2nd vaccination with PBS, RBD, RCP, Alv, and GCR vaccines with hypodermic injection.

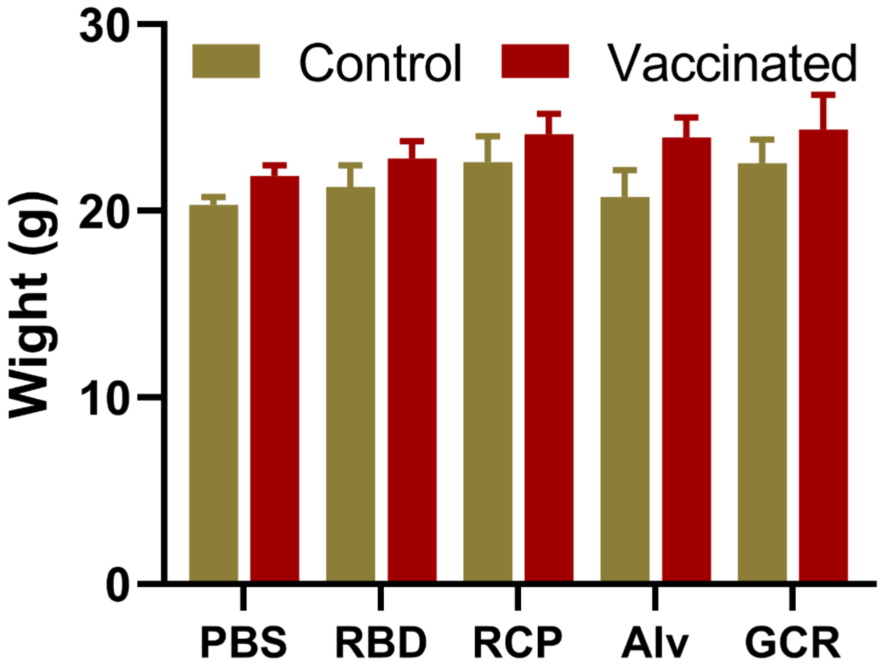

Figure S5. Gating strategy for splenic T cells. (A) Gating scheme for the identification of cytokine-expressing $CD4^+$ and $CD8^+$ T cells. (B) Gating scheme for the identification of T_{cm} in $CD4^+$ and $CD8^+$ T cells.

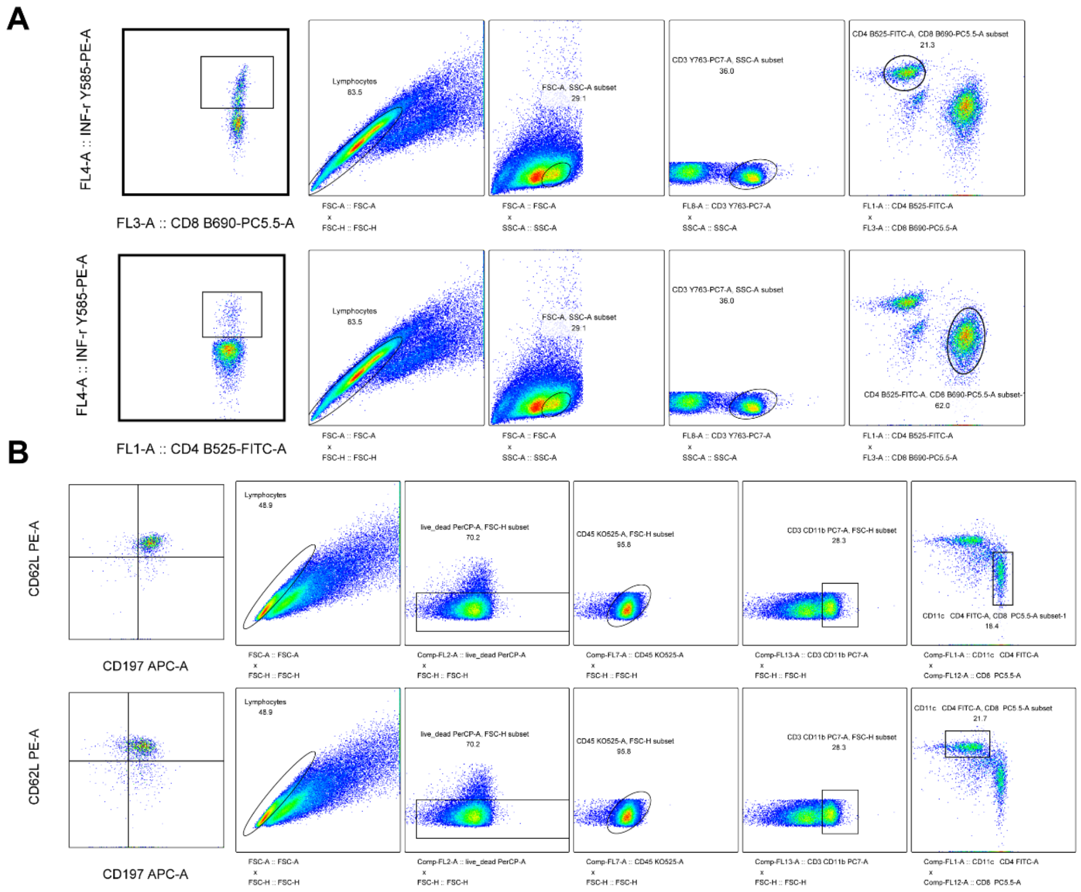

Figure S6. Type 1 cellular cytokines concentration in spleen lymphocytes culture supernatant after 24 h incubation with RBD peptide pools.

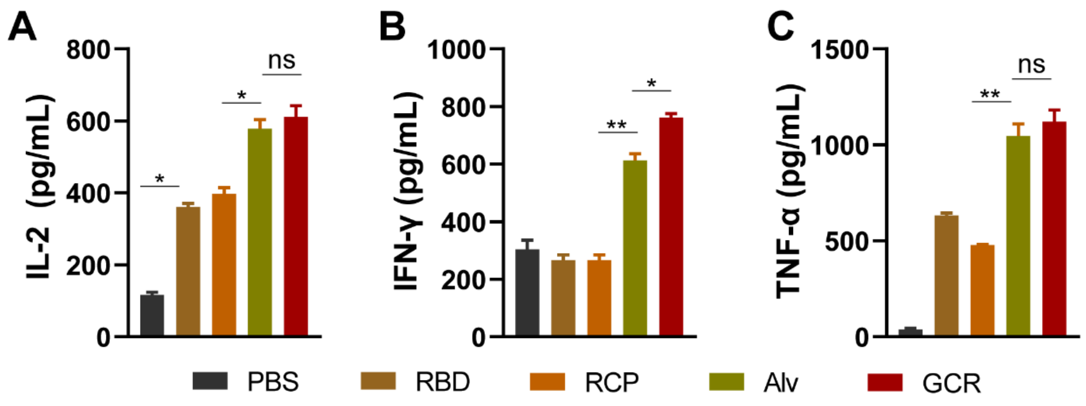

Figure S7. Mouse skin healing status after MGCR microneedle administrated for 30 seconds

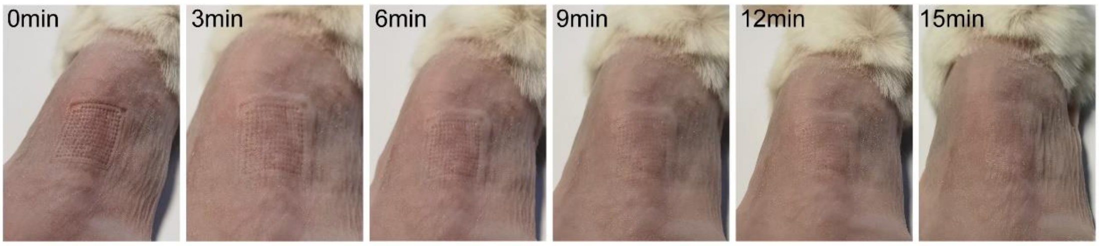

Figure S8. *In vivo* distribution of the MGCR in different mouse tissues after administrated 24 hours.

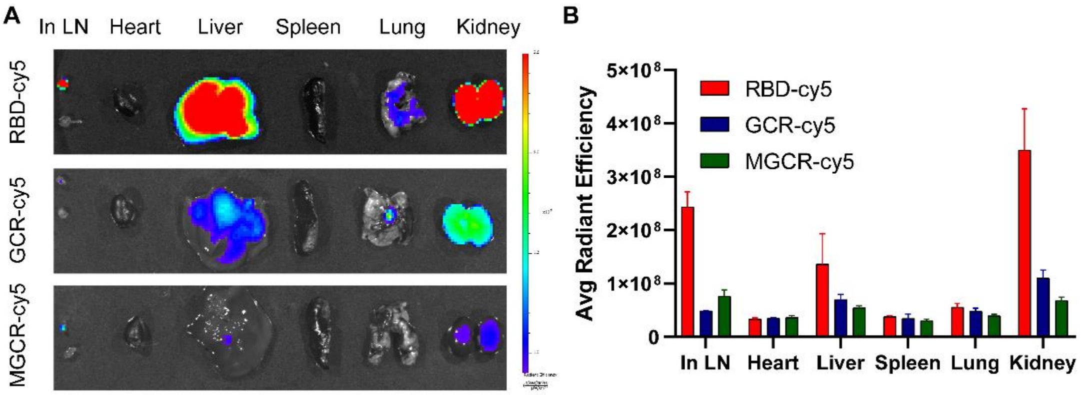

Figure S9. The representative flow cytometry spectrum and quantitative statistics of type 1 cellular cytokines expression in MGCR microneedle vaccination experiment.

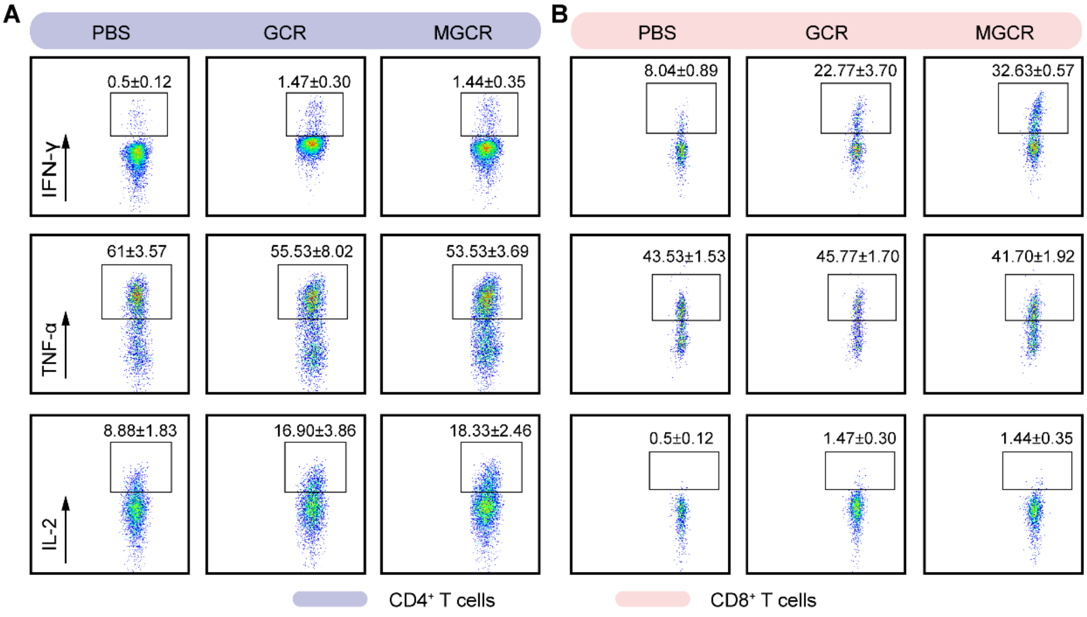

Supplement: Supplementary file 1 — Supplementary figures and tables. [file thnov13p4821s1.pdf]
